# Retraction Note: Haplotype analysis on correlation between transcription factor 7-like 2 gene polymorphism and breast cancer risk

**DOI:** 10.1186/s12885-024-12097-w

**Published:** 2024-03-21

**Authors:** Yang Wang, Xiaojuan Men, Yongxue Gu, Huidong Wang, Zhicai Xu

**Affiliations:** Department of Galactophore Surgery, Weifang People’s Hospital, No.151 Guangwen Street, Kuiwen District, 261041 Weifang, Shandong Province People’s Republic of China

**Retraction Note**: ***BMC Cancer 21, 885 (2021)***

10.1186/s12885-021-08571-4

The Editor has retracted this article because of substantial overlaps with article [1] by different authors that was under submission at the same time. The authors did not respond to a request to explain the overlaps and to provide evidence that an ethics approval had been obtained before the commencement of the study. The authors did not respond to correspondence from the Editor about this retraction.

## References

[1] Xue P, Cao H, Ma Z, et al. Transcription factor 7-like 2 gene- smoking interaction on the risk of diabetic nephropathy in Chinese Han population. *Genes and Environ*. 2021;43:26. 10.1186/s41021-021-00194-2. 10.1186/s41021-021-00194-2 PMC8244137 34193317

